# Fc Receptors for Immunoglobulins and Their Appearance during Vertebrate Evolution

**DOI:** 10.1371/journal.pone.0096903

**Published:** 2014-05-09

**Authors:** Srinivas Akula, Sayran Mohammadamin, Lars Hellman

**Affiliations:** Department of Cell and Molecular Biology, Uppsala University, The Biomedical Center, Uppsala, Sweden; California State University Fullerton, United States of America

## Abstract

Receptors interacting with the constant domain of immunoglobulins (Igs) have a number of important functions in vertebrates. They facilitate phagocytosis by opsonization, are key components in antibody-dependent cellular cytotoxicity as well as activating cells to release granules. In mammals, four major types of classical Fc receptors (FcRs) for IgG have been identified, one high-affinity receptor for IgE, one for both IgM and IgA, one for IgM and one for IgA. All of these receptors are related in structure and all of them, except the IgA receptor, are found in primates on chromosome 1, indicating that they originate from a common ancestor by successive gene duplications. The number of Ig isotypes has increased gradually during vertebrate evolution and this increase has likely been accompanied by a similar increase in isotype-specific receptors. To test this hypothesis we have performed a detailed bioinformatics analysis of a panel of vertebrate genomes. The first components to appear are the poly-Ig receptors (PIGRs), receptors similar to the classic FcRs in mammals, so called FcRL receptors, and the FcR γ chain. These molecules are not found in cartilagous fish and may first appear within bony fishes, indicating a major step in Fc receptor evolution at the appearance of bony fish. In contrast, the receptor for IgA is only found in placental mammals, indicating a relatively late appearance. The IgM and IgA/M receptors are first observed in the monotremes, exemplified by the platypus, indicating an appearance during early mammalian evolution. Clearly identifiable classical receptors for IgG and IgE are found only in marsupials and placental mammals, but closely related receptors are found in the platypus, indicating a second major step in Fc receptor evolution during early mammalian evolution, involving the appearance of classical IgG and IgE receptors from FcRL molecules and IgM and IgA/M receptors from PIGR.

## Introduction

**Figure 1:**
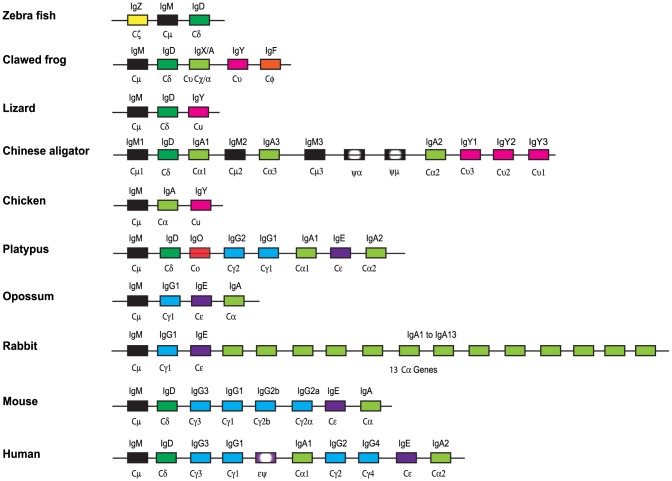
The immunoglobulin heavy chain locus of a panel of selected vertebrates from fish to humans. The locus depicts every single gene as a block and without individual exons. The figure is not to scale and the genes have been color coded; IgM in black, IgD in dark green, IgA and IgX in light green, IgY in magenta, IgG in blue, IgE in purple, IgO in red, IgZ in yellow, IgF in orange and pseudogenes in shaded grey or purple.

Immunoglobulins (Igs) are only found in jawed vertebrates and there are strong indications that the complexity of the adaptive immune system has increased gradually during vertebrate evolution. The effector functions of the Igs have separated into different Ig classes thereby increasing the regulatory potential of the immune system. Mammals express up to six different Ig classes: IgM, IgD, IgG, IgE, IgA and IgO, and the total number of isotypes can sometimes exceed 15 (Figure 1) [1]. Of these six Ig classes only IgM and IgD have been found in fish, which tend to have only two to three Ig classes and isotypes. In fish, the list of Ig classes now also includes IgW, IgNAR, IgT and IgZ [2]–[6]. In general, amphibians have four to five classes of Igs: IgM, IgD, IgA/IgX, IgY and one additional class named IgF or IgP [7], [8]. Neither IgA nor IgY is found in fish and IgG and IgE have not been identified in reptiles, amphibians or birds, suggesting they are unique for mammals [9]. Birds have only three Ig classes: IgM, IgA and IgY (Figure 1). However, this low number is most likely a result of a loss of isotypes as massive losses and re-expansion of genes and gene families have occurred in birds [10]. Interestingly, other members of the reptile lineage have instead experienced massive expansions like the Chinese alligator, which has 10 isotypes, although neither IgG nor IgE [4]. All major classes in placental mammals are also present in monotremes, the egg-laying mammals. The platypus has been shown to express six classes and eight different isotypes: IgM, IgG1, IgG2, IgA1, IgA2, IgE, IgD and IgO (Figure 1) [1], [11]–[14]. Marsupials, as represented by the American opossum have IgM, IgG, IgA and IgE, but no IgD gene [15], [16]. The gene for IgD has probably been lost in this lineage (Figure 1) [17]. These findings indicate that the number of Ig classes and isotypes has increased during vertebrate evolution from two to three in fish, to sometimes more than fifteen in mammals (Figure 1). The most important steps in this increase have probably been the duplication of IgM forming the early ancestor of IgY. This seems to have occurred at the emergence of the tetrapods, The second step was most likely a duplication of IgM, forming the ancestor of IgA/X. This isotype is also first observed in amphibians. The third major step was the duplication of IgY forming IgG and IgE. However, losses of classes and isotypes have also occurred as exemplified by the loss of IgD in birds and marsupials. Interestingly, the chicken has only one light chain isotype, whereas mammals have two and many fish species have three, indicating that birds have lost two light chain loci. The increase in the number of different Ig classes and isotypes observed in many tetrapods has likely been accompanied by a similar increase in isotype-specific receptors. However, how this increase has occurred is still only partly understood.

**Figure 2:**
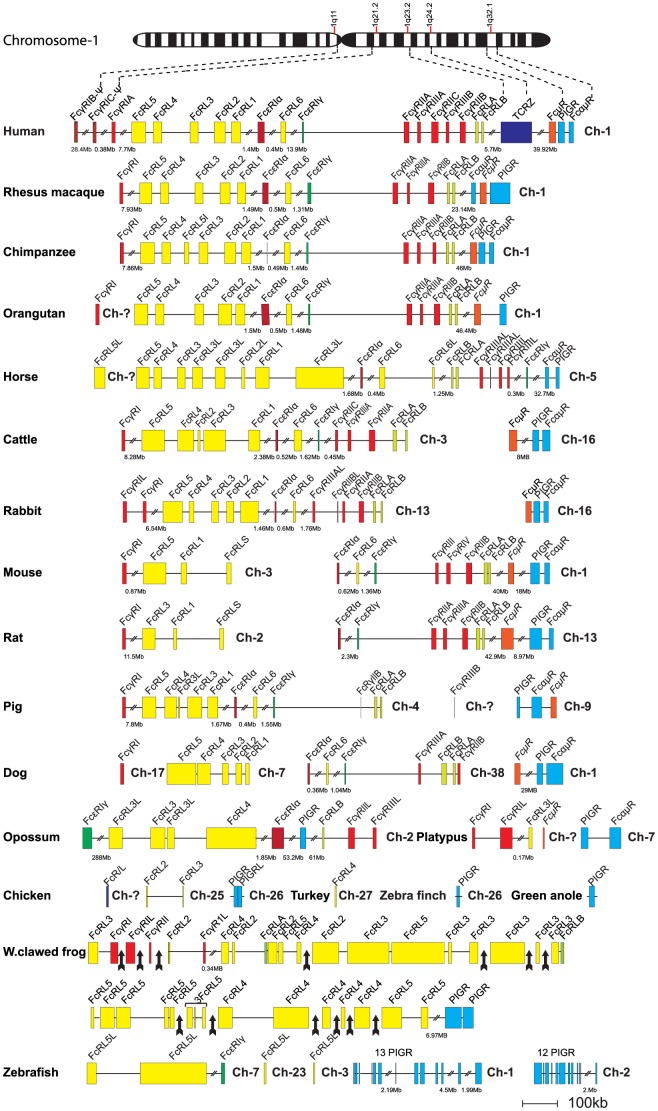
The major Fc receptor gene loci in a panel of different vertebrates. Each horizontal line corresponds to a chromosome on which different Fc receptor genes are located. Genes are color coded. The Fc receptor-like (FcRL) genes are shown in yellow except the FcRLA and B that are in light green, classical IgG receptors in red (pseudogenes in striped red), IgE receptor in dark green, the IgM receptor in orange, the poly-Ig receptor (PIGR) and the IgA/IgM receptor in blue. The figure contains the genes identified for the following animal species: Zebrafish (*Danio rerio*), Western clawed frog (*Xenopus silunarana* and *Xenopus tropicalis*), Chicken (*Gallus gallus*), Turkey (*Meleagris gallopovo)*, Zebra Finch (*Taeniopygia guttata*), Green anole (*Anolis carolinennsis*), Platypus (*Ornithorhynchus anatinus*), Opossum (*Monodelphis domestica*), Rat (*Ratuus norvegius*), Mouse (*Mus musculus*), Horse (*Equus caballus*), Rabbit (*Oryctologus cunicullus*), Pig (*Sus scrofa*), Dog (*Canis lupus familaris*), Cattle (*Bos taurus*), Orangutan (*Pongo abelli*), Chimpanzee (*Pan troglodytes*), Rhesus macaque (*Macaca mulatta*), Human (*Homo sapiens*).

**Figure 3:**
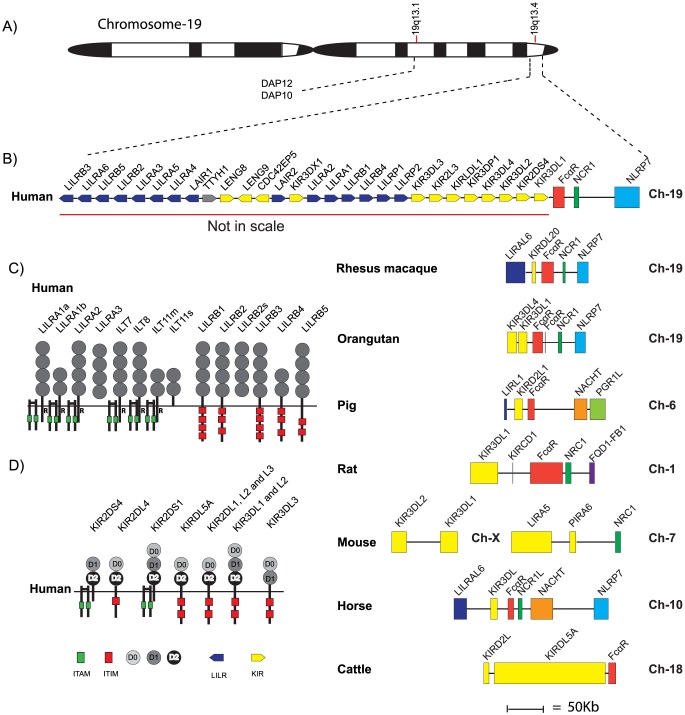
The FcαR locus and neighboring genes. Panel A shows a schematic drawing of chromosome 19, panel B a schematic drawing of the LRC locus, panel C the domain structure of the LILRs of this locus (an ‘R’ represents arginine in the transmembrane region) and panel D the domain structure of the KIRs. In the detailed map of the region encoding the IgA receptor each horizontal line corresponds to a chromosome on which different Fc receptor genes are located. Genes are color coded. The IgA receptor in red, the KIRs and LILRs in yellow, the neighboring genes that are used as markers for the chromosomal region, NCR1 in dark green, the NACHT in orange, the NLRP7 in light blue, the PGRL1 in light green and the FQD1-FB1 in dark blue. The overall structure of the LRC locus and the protein domain structures have been adopted from Espeli et al 2010 and Brown et al 2004 [45], [68].

Humans and mice have a complex set of Fc-specific receptors that match the number of different Ig-isotypes and subclasses (Figures 2 and 3). Four major types of classical Fc receptors for IgG: FcγRI, FcγRII, FcγRIII and FcγRIV, which show varying affinities for the four IgG isotypes, have been identified [18], [19]. Additionally, one high-affinity receptor for IgE (FcεRI), one for both IgM and IgA (FcαμR), one for IgM (FcμR) and one receptor for IgA (FcαRI) are found in almost all placental mammals studied [20]–[22]. The α chains of these receptors, which are the Ig-binding subunits, are all related in structure and it's likely they originate from one or a few common early ancestors via successive gene duplications [23]. In humans, all α chains of the IgG and IgE receptors have two extracellular Ig-like domains except for FcγRI, which contains three such domains. The opossum has recently been shown to have all of the classical IgG and IgE receptors except FcγRI, the high-affinity receptor for IgG, indicating that the two-domain receptors were the first to appear during mammalian evolution [24]. All of the IgG and IgE receptors, except one variant of FcγRIII in humans, FcγRIIIB, also have a transmembrane region that anchors the receptor in the membrane [18]. FcγRIIIB is a glycosylphosphatidylinositol (GPI) anchored activating receptor that has no cytoplasmic tail. In some of these receptors the cytoplasmic tail region is mediating the signal to the cell. However, for many of them the signaling function is found in another subunit of the receptor, the γ chain [25]. This non Ig-domain containing signaling subunit is a member of a small family of related molecules including the T-cell receptor (TCR) zeta chain, DAP10 and DAP12 [26]–[29]. The latter two proteins serve as signaling components of the NK cell receptor and related Ig-domain containing receptors [29]. The biological functions of the FcRs are regulated by immunoreceptor tyrosine-based activation motifs (ITAMs) and immunoreceptor tyrosine-based inhibitory motifs (ITIMs), which activate or inhibit the cellular function, respectively [18], [20]. These motifs are located in the cytoplasmic part of the γ chain and in the case of FcγRIIB, in the cytoplasmic part of the α chain. FcRs with ITAMs elicit cell activation, endocytosis and phagocytosis, whereas receptors with ITIMs have an inhibitory effect on cell activation. The FcεRI also has a separate β chain, which most likely has a role in enhancing signaling, given it has one cytoplasmic ITAM motif [26]. The β chains show no homology to the α chains, are located on another chromosome, and are members of the MS4A family, which includes molecules like CD20, HTm4 and at least 18 other members in the human genome [30].

The FcμR has only one Ig domain and is relatively distantly related to the other receptors. Its cytoplasmic part has a well conserved Ser-Tyr rich region instead of an ITIM or ITAM, and is located in the same chromosomal region as the IgG and IgE receptors [31].

In addition to all the receptors described above where the α chains have Ig-like domains, there are at least two FcRs of a totally unrelated nature, the IgD and the low-affinity IgE receptor, also named CD23. Both of these are lectins and will not be discussed more in this communication.

In addition to the classical FcRs, a new family of related receptors was discovered upon the completion of full genome sequences from a number of mammalian species [32], [33]. Eight different such Fc receptor-like (FcRL) genes have been identified in the human genome, FcRL1-FcRL6 as well as FcRLA and FcRLB. Duplications of these genes are denoted FcRLxL, where *x* represents one of the original FcRL1-6. Interestingly, the number of extracellular Ig-like domains differs quite significantly between these receptors; from nine in FcRL5 to three in FcRL1 and 6. In humans, all of them are located in the same chromosomal region as the majority of the classical FcRs. In the same region we find FcRLA and FcRLB. Both of these members lack a transmembrane region and are found in the cytoplasm of the cell [32], [33]. The ligands for these potential receptors have been a mystery until very recently, where FcRL4 and 5 were found to bind IgA and IgG, respectively, and FcRL6 was found to interact with MHC class II molecules [34]–[36]. However, the other FcRL molecules still await identifying ligands and functions. In contrast to the majority of the classical FcRs, the FcRL molecules seem to have an overrepresentation of ITIMs in their cytoplasmic tails, which indicates that they primarily have a dampening function during immune responses. Interestingly, and in accordance with such a hypothesis, genetic studies have indicated involvement of variants of FcRL3 in autoimmune disease [37].

The poly-Ig receptor (PIGR) is one additionally related receptor that is also found in the same chromosomal region as the classical FcRs, and is the transport receptor for IgA and IgM [21], [38]. This molecule has two to five extracellular Ig-domains depending on the species [21], [39]–[41].

Other Ig-domain containing receptors like the natural killer Ig-like receptors (KIRs), the leukocyte Ig-like receptors (LILRs) and the leukocyte-associated Ig-like receptors (LAIRs), are also distantly related to the classical FcRs and the FcRL receptors [42], [43]. The mouse homologs of the LILRs in humans are named PIRs. The ligands for the KIRs and LILRs of mammals are primarily MHC class I molecules [44], [45]. Interestingly, the FcαR shows higher sequence relatedness (35%) to the KIRs and LILRs than to the classical FcRs (only 20% identity) and is also located within the LRC on chromosome 19 in humans together with the KIRs and LILRs [45], [46]. Homologs to the genes in the LRC gene complex have been found in early ancestors of vertebrates, the tunicates [47]. A large number of related genes have also been identified in several species of fish including catfishes, indicating an early appearance of these receptors during vertebrate evolution [48]. The LRC genes in chicken are named Ig-like receptors (CHIRs) [49], which all have one or two extracellular Ig-like domains.

Generally, Ig domains have been classified as V, C1, C2 or I domains depending on features such as the spacing of cysteine bridges and the number of beta sheets. V domains are generally found in variable regions of Igs and TCRs as well as in CD2 CD4, CD80 and CD86. C1 domains are found in constant regions of Igs, TCR and in MHC class I and II. C2 domains are found in CD2, CD4, CD80, VCAM and ICAM, and I domains are found in VCAM, ICAM, NCAM, MADCAM and numerous other diverse protein families (EMBL-EBI InterPro). All Ig domains of CHIRs, KIRs, LILRs, the FcRL and the classical FcRs are classed as C2 domains and the Ig domains of the PIGR, IgM, and IgA/IgM receptors are V type domains [49], [50].

An interesting possibility is that all of these Ig-domain receptors within the FcR locus and the LRC originate from a common ancestor. These two loci may be the result of the genome duplications that most likely occurred during early vertebrate evolution [47], [51]. Due to fact that receptors from both the LRC and the FcR loci can be positive and negative regulators, depending on the presence of ITIMs or ITAMs, they have been named paired receptors [52].

In order to obtain a more detailed picture of the appearance of all these receptors and how their appearance correlates to the increase in Ig-isotype repertoire, we used bioinformatics to screen for related receptors in a panel of vertebrate genomes. The results presented in this communication show that the transport receptor for IgA and IgM, the PIGR, the FcRL family and the signaling component the common γ chain appeared with the bony fish, indicating a major event in the evolution of these receptors at the base of the bony fish. A second major event in FcR evolution apparently occurred during early mammalian evolution with the appearance of the IgM, the IgA/M receptors and the formation of a separate subfamily of FcRL molecules which today includes all of the classical receptors for IgG and IgE.

## Materials and Methods

Identifying the gene loci of FcRs and their sequences was conducted using NCBI (National center for Biotechnology Information) and Ensemble databases (http://www.ncbi.nlm.nih.gov/) (Ensemble http://www.ensembl.org/index.html). Human FcR protein sequences were initially used as query sequences using TBLASTN (translation BLAST, Translate Basic Local Alignment Search Tool). Later sequences obtained from the studied species were used to screen for potential additional homologous genes to complete the picture. The gene loci were identified from the resulting data and the sizes of the genes, including the distance between the neighboring genes, were calculated and used to construct maps in scale of the loci. The gene arrangement and receptor domain figures were made in Adobe illustrator. The gene accession numbers used in constructing the figures are listed in Supporting Information S1.

### Phylogenic analysis

In order to study the relationship between the various sequences, the entire coding region of the proteins were run in multiple alignments using MAFFT. For phylogeny analyses we used the PHYLIP, MrBayes, MEGA and CLUSTALW programs [53], [54], [55].

#### Alignments

The selected Fc receptors of vertebrates were aligned using version 7 of MAFFT (http://mafft.cbrc.jp/alignment/server/), using the E-INS_I strategy for optimal results for sequences with conserved motifs and carrying multiple domains, with default parameters [56].

#### Phylogeny

Clustal X: MAFFT alignment files was imported into Clustal X version 2.0 [55] to generate a neighbor joining tree with 100 bootstrap replicates. The tree file was viewed in Fig tree (http://tree.bio.ed.ac.uk/software/figtree/). Phylip: The Phylip package (v3.69)(http://evolution.genetics.washington.edu/phylip.html) was used for constructing Maximum-likelihood and Distance trees [53]. For the distance method PROTDIST, FITCH (JTT matrix Model without using an out-group species) were used. For the bootstrap analyses SEQBOOT, PROTDIST, NEIGHBOR and CONSENSE was used to generate 100 replicate data sets from the Phylip package (v3.69). For Maximum-likelihood trees using PROML and viewed in Fig tree. MrBayes: The phylogenetic analysis was performed using a Bayesian approach as used in MrBayes version 3.2. [54]. Markov Chain Monte Carlo (MCMC) analysis was used to approximate the posterior probabilities of the trees. The phylogenetic tree was drawn in FigTree 1.3.1. MEGA (Molecular Evolutionary Genetics Analysis Version 5.2.2)[57]. Multiple alignment file obtained from MAFFT converted into MEGA format and phylogenetic analysis was performed using Neighbor joining and Maximum-likelihood methods in both methods using boot replications was 500. The distances were calculated by JTT matrix method.

The individual domains of the FcR for a few selected key species were also analyzed for their relatedness using the MEGA Neighbor joining and MEGA Maximum-likelihood methods. The domains were color coded according to a pattern that previously been used by several other labs [24], [32], [58]. The results are shown in Figure S4.

## Results

Human FcR protein sequences were used as query sequences to identify similar sequences in a large panel of vertebrate genomes in the NCBI and Ensemble databases using the TBLASTN (translation BLAST) algorithm. The different gene loci were identified, the size of the genes and the distances between genes were calculated and used to produce maps drawn to scale of these chromosomal regions as presented in figures 2 and 3.

### Mammals

All the primates that we have analyzed (human, macaque, chimpanzee and orangutan) have their classical FcRs and the FcRL molecules (except FcαR), on chromosome 1 (Figure 2). However, these genes are not found in a small well-defined region but spread over a large portion of chromosome 1. In contrast, the IgA receptors are located together with some of the NK cell receptors, the KIRs, and the LILRs on chromosome 19 (Figure 3). Interestingly, homologs to the human IgA receptor were only found in placental mammals and not in any of the other vertebrates, indicating a relatively late appearance. The FcαR is located on chromosome 19 in humans, macaques and orangutans, on chromosome 6 in pigs, chromosome 1 in rats, chromosome 10 in horses and on chromosome 18 in cattle (Figure 3). However, no IgA receptor has been identified in the mouse genome. The region, which in other placental animals harbors the FcαR, is broken up into two fragments in mice. These products have ended up on different chromosomes, by a process that most likely resulted in the loss of the FcαR gene (Figure 3).

Within the cluster of genes on chromosome 1 in primates, which contains all the classical receptors and the FcRL molecules (except the specific IgA receptor), a few differences are found between species in gene numbers and organization. In the human genome there is a duplication involving the low-affinity receptors for IgG (FcγRII, and FcγRIII), as well as two additional genes related to the high-affinity receptor for IgG, FcγRI. The new genes for the low-affinity receptors have been named FcγRIIC and FcγRIIIB and the new high-affinity receptor genes, FcγRIB and FcγRIC. These two latter genes are inactivated pseudogenes (Figure 2). The chimpanzee has one extra FcRL5L receptor gene, which is probably the result of a recent gene duplication. In the orangutan, FcγRI is still on an unassigned contig and therefore cannot definitely be assigned to chromosome 1.

In the horse, all the classical FcRs, the FcRL1-6 and FcRLA and B are also found on one chromosome, here on chromosome 5. However, FcγRI has not yet been identified, which may be due to an incomplete genome. In many of the placental mammals this locus has been broken up into several fragments, which have ended up on different chromosomes. This indicates that in many placental mammals, frequent translocations in this region have occurred (Figure 2). In rats and mice, the loci are separated into two fragments. In the rat, FcγRI, FcRL1 and FcRLs are on chromosome 2, whereas the intracellular proteins FcRLA and FcRLB, the PIGR, the FcαμR, the FcμR and the FcεRIα are on chromosome 13 (Figure 2). In the mouse, FcγRI, FcRL5, FcRL1 and FcRLS are on chromosome 3. The remaining receptors are on chromosome 1 (Figure 2). FcRLS is a soluble mosaic protein containing a scavenger receptor cysteine rich type B domain (SRCR) [32] .

Interestingly, the rabbit has all the genes of this locus on chromosome 13, except for FcαμR, the FcμR and PIGR, which are located on chromosome 16. A duplication of the high-affinity receptor for IgG, FcγRI has also occurred in the rabbit genome (Figure 2). Cattle show a similar organization as rabbits with FcαμR, the FcμR and PIGR on one chromosome, 16, whereas the other receptors are on chromosome 3. Pigs also show a similar organization with FcαμR, FcμR and PIGR on chromosome 9 and the other receptors on chromosome 4. However, one of the genes FcγRIIIB is still on an unassigned contig (Figure 2). Dogs show the most complicated pattern with these genes separated onto four different chromosomes. The FcRL receptors are on chromosome 7, the intracellular proteins, FcRLA and FcRLB, and the classical FcRs on chromosome 38, FcγRI on chromosome 17 and FcμR, FcαμR and PIGR on chromosome 1 (Figure 2).

In marsupials almost all of the different families identified in humans are present. In the opossum, all the classical receptors, FcγRIIIL, FcγRIIL, FcεRI, the FcRL receptors, FcRL3L, FcRL3, FcRL4 and the intracellular proteins FcRLA, FcRLB, as well as the PIGR, and FcεRIγ are found on chromosome 2 (Figure 2). However, no copy of the three-domain receptor for IgG, the FcγRI was identified as previously also shown by Fayngerts et al. [24]. The opossum genome sequence has gone through peculiar changes and sequences that appeared in the draft genome have sometimes disappeared in the final assembly. This makes a detailed analysis complicated as genes may have been deleted by mistake.

The picture gets more complicated when analyzing the monotremes. Despite a relatively incomplete platypus genome, two genes with homology to the high-affinity IgG receptor, the FcγRI are found (Figure 2). These genes FcγRIα, FcγRIαL, plus two genes with homology to FcRL3L and the PIGR are found on chromosome 7. In the same region there are pieces of two, two-domain receptors, indicating that both two- and three-domain receptors were already present during early mammalian evolution. No copy of the the IgA receptor was found in the platypus genome but a copy of the FcµR and very recently the FcαμR was found indicating a slightly earlier appearance of these latter receptors, possibly during early mammalian evolution.

### Reptiles and Birds

Reptiles and birds add a layer of complexity to the picture. There are only a few genomes that are close to completion and in birds, massive losses and re-expansions of genes and gene families have occurred, which complicates the analyses even more [10]. In the chicken genome there are no close homologs to the classical FcRs, and no FcRLA or B. Instead, the chicken has undergone a massive expansion of KIR-related CHIRs found in the LRC region in mammals, where a large number of them are found on micro chromosome 31 [49]. Of the other receptors found on chromosome 1 in primates, the PIGR, one FcRL2L gene as well as one additionally related sequence FcR/L are found (Figure 2). The FcRL2 and FcRL3 are situated on chromosome 25, two copies of the PIGR are located on chromosome 26 and FcR/Lon a yet unknown chromosome in the chicken. In the incomplete turkey genome there is a FcRL4-related gene on chromosome 27. The zebra finch has one copy of the PIGR on chromosome 26. Looking at a reptile, the green anole, there is a contig that harbors the PIGR (Figure 2).

### Amphibians

**Figure 4:**
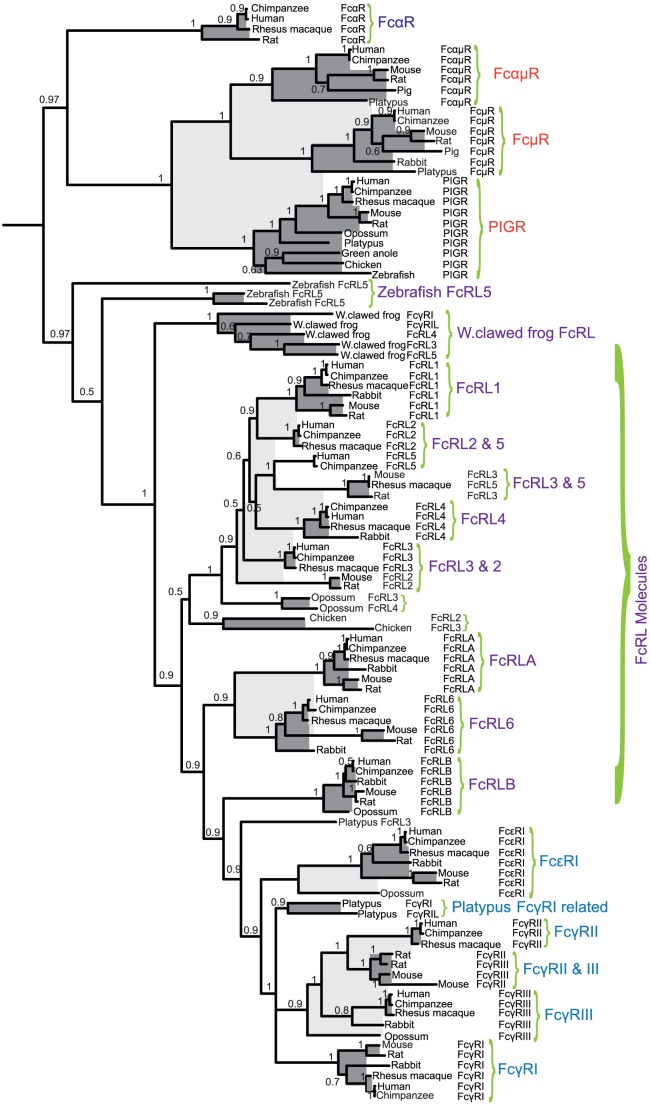
A phylogenetic tree of Fc receptor sequences from a panel of vertebrates analyzed with the MrBayes program. The Maximum-likelihood tree was based on the Bayesian methods of phylogenetic interference. Robustness of nodes was tested with the posterior probabilities based on MCMC analysis as implemented in the MrBayes program. Node supported posterior values are given in the phylogenetic tree. Groups of genes that are more closely related, thereby forming a sub-branch, are indicated by light or dark grey shading to make them more easily visible.

**Figure 5:**
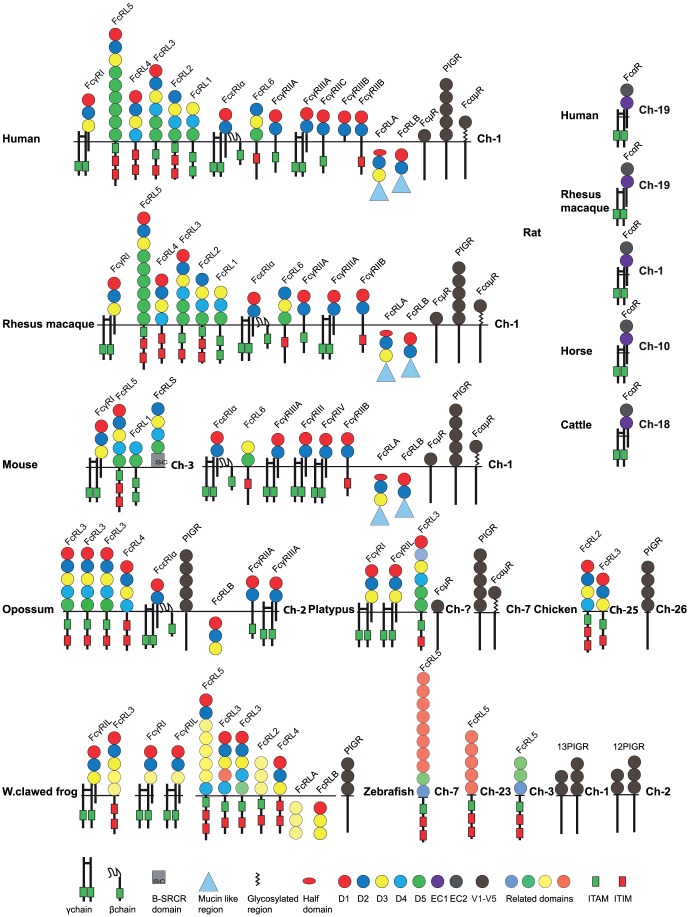
A summary of domain structures and signaling motifs of the various vertebrate Fc receptors. The Ig-like domains are depicted as filled circles with color-coding according to the similarities in sequence based on phylogenetic analyses [24], [58]. The domain type D1, D2, D3, D4 and D5 show a relatively conserved pattern in most tetrapods and have therefore been color-coded in red dark blue, yellow, light blue and green. A phylogenetic analysis of all the individual domains presented in figure 5 and a few additional receptors are presented in supplementary figure S4. The color-coding in figure 5 is based on this supplementary figure. The extracellular regions, the transmembrane regions and cytoplasmic tails are not to scale in order to show the positions of potential signaling motifs like ITAMs (green boxes) and ITIMs (red boxes), which regulate the biological function the Fc receptors. Non-consensus ITIMs are also indicated as boxes with half red half white. Some of the intracellular proteins contain C-terminal mucin-like regions that are depicted as blue triangles.

In the Western clawed frog (*Xenopus tropicalis*), there are three gene copies of a receptor, showing similarity to the the α chain of FcγRI (Figure 2). All three copies have three extracellular Ig-like domains and have been named FcγRI and FcγRIL and FcγRIL. However, they are relatively distantly related and have therefore likely appeared via two successive gene duplications early in amphibian evolution (Figure 4). Additionally, one copy of the PIGR, FcRLA and B and a very large number of FcRL genes were found (Figure 3). Very recently a copy of a two-domain receptor appeared in the current version of the *Xenopus* genome. A massive expansion of the FcRL genes has resulted in at least 31 genes, which all show similarity to the human FcRL genes 3, 4 and 5 (Figures 2 and 5). The clusters of FcRL3L, FcRL4L, FcRL5L and the FcγRI receptors are found on different scaffolds. The fact that the genome sequence is incomplete and that the genes are scattered over a large number of scaffolds makes the analysis difficult, where the likelihood that more genes will be found is high.

### Bony fish

In the zebrafish only the PIGRs, one FcεRIγ chain gene and four FcRL5L genes are found. In zebrafish, the PIGR and FcεRIγ chain are located on chromosome 2, and the four FcRL5L genes are shared on chromosome 3 and 7, with two on each (Figure 2). Interestingly, a massive duplication of the PIGRs has occurred in the zebrafish. Thirteen very similar PIGR genes are found in a cluster on chromosome 1 and twelve on chromosome 2 (Figure 2). The 25 copies in the zebrafish all have two or three extracellular Ig-like domains and they are no greater than 20% different in amino acid sequence (data not shown), indicating a relatively recent expansion.

### Cartilagous fish and lampreys

The genomes of a number of shark species (elephant, ground, requiem, carpet, nurse, grey, cat, dogfish, tiger, horn and angel) and the lamprey were analyzed for genes homologous to the various receptors. However, no homologs of either the PIGRs, the FcεRIγ chain, the FcRL or the classical receptors were found even after extensive screening using both mammalian and fish sequences, indicating that all of them first appear with the bony fish. Interestingly, no direct homologs to the LRC genes with only C2 domains were found in any of the shark genomes or the lamprey also indicating that they appear with the bony fish.

### Phylogenetic analysis

All the FcR sequences represented in figures 2 and 3 were analyzed for sequence relatedness using Neighbor-joining, Distance, and Maximum-likelihood methods. In this study we used the entire sequence of the receptors. The MrBayes tree is presented in figure 4, whereas the Neighbor-joining analysis, Maximum likelihood and the Distance trees are presented as supplementary figures S1, S2 and S3. The three V domain-containing receptors (the IgM, the PIGR and the IgM/IgA receptors) are the most distantly related members of this group of proteins, which spontaneously form an out-group. Regarding the classical receptors, all of the IgG and the IgE receptors form separate sub-branches but cluster together in one major branch, including the three-domain receptor FcγRI. The FcRL sequences are more closely related to the IgG and IgE receptors than to the IgM and IgA receptors. In several of the trees some of the platypus sequences cluster with the classical Fc receptors, in particular the FcγRI sequences. This latter finding indicates that the platypus sequences are actually the first step in the formation of a separate group of receptors that now constitute the classical IgG and IgE receptors.

Interestingly, the Western clawed frog sequences form separate branches in the various trees, indicating that the individual members have appeared after the separation from the other vertebrate lineages. Another prominent finding is that all the classical Fc receptors cluster with FcRL sequences, often closest with FcRLA and B or as in the case with the three platypus FcγRI-like sequences, with platypus FcRL3 (Figure 4). All three, three-domain receptors in the Western clawed frog genome that were identified during the screening with the human FcγRI sequence also cluster with frog FcRL3, 4, 5 and the FcRLA and B (Figures 4, S1, S2 and S3).

### Domain structure

Individual domains of the majority of the FcRs from a few selected key species were analyzed for their relatedness by a phylogenetic analysis using Neighbor joining and Maximum likelihood methods. The result from the two analyzes were almost identical and therefore one is found as Figure S4. In total, 333 domains were included in the analysis. In Figure 5, the result from this analysis has been translated into a figure showing the number of domains and their relationship by color-coding the individual domains. Domain type 1 is coded in red. The zebrafish FcRL molecules have domains that are related to the domain 1 of mammals and are therefore coded in a lighter shade of red. Domain type 2 is shown in dark blue, domain type 3 in yellow and type 4 and 5 that are closely related and shown in light blue and green, respectively. A number of the Xenopus FcRL domains are closely related to the type 3 domains of mammals and are therefore coded in yellow. The V type domains of the PIGR, the IgM and the IgA/IgM receptors form a separate branch in the phylogenetic tree and are shown in grey in figure 5. The summary figure 5 shows the number of extracellular Ig-like domains and the intracellular region with ITAMs and ITIMs. The question of ITAMS and ITIMs is not easy to resolve as several motifs are non-canonical ITIMs with similarity to the consensus. We have therefore depicted consensus and closely related sequences separately in figure 5. Interestingly, many of the FcRL molecules lacked consensus ITIMs and in Xenopus some FcRL5 molecules had up to 2 consensus ITIMs whereas other members of the same subfamily had no ITIMs, or even closely related sequences. Due to the large number of FcRL genes in Xenopus, a few of them are shown in figure 5 and a number of additional members are depicted in figure S5.

In addition, the domain analysis showed that the D1, D2 and D3 domain types are all present in the same order in FcγRI and FcRL3, 4, 5 and A and B. The D1 and D2 domain types are also present in the same orientation in the two domain FcRs in mammals, indicating a close relationship in structure of FcRL and the classical FcRs in mammals (Figure 5).

## Discussion

Based on our screening of a large panel of vertebrate genomes, we can now conclude that the first components to appear within the FcR locus are the PIGRs, the FcRL molecules and the signaling subunit, the γ chain as they are found in all bony fish species analyzed. Interestingly, no genes with clear similarities to either of these genes/gene families were found in cartilagous fish and lamprey, indicating that they appeared at the base of the bony fishes. The receptors homologous to the mammalian members of the LRC locus, the KIRs and LILRs seems also to appear with the bony fish as no clear homologs with only C2 domains can be found in sharks or lamprey. This indicates a major evolutionary step in the emergence of a complex adaptive immune system of jawed vertebrates occured at the base of the bony fish. Immunoglobulins and T-cell receptors first appeared with the jawed vertebrates through the introduction of the recombination activating genes (RAGs) and the formation of a split V gene facilitating variability generation by RAG mediated recombinations [59]. The second major step may then have been the appearance of the various Fc receptors and the KIRs and LILRs at the base of the bony fish.

Interestingly, relatively few FcRs and FcRL genes are found in fishes and chicken. Instead there has been an extensive expansion of the LRC locus genes. A very large number of LRC members are found in the channel catfish and also more than 100 copies of the chicken homologs, the CHIRs, are found on the micro chromosome 31 in chickens [48], [49]. Several of the CHIRs on micro chromosome 31, including CHIR-AB1, have been identified as a receptor for IgY but none have been found to bind either IgM or IgA [49], [60]. An additionally related single gene at the tip of chromosome 20 has four extracellular C2-like Ig domains, which has been shown to be a high-affinity receptor for IgY [61]. This gene has been named ggFcR and requires the common FcR γ chain for signaling [61]. This gene shows the closest homology to the FcRL molecules and is possibly one of a few remnants of this locus in birds. Therefore in chickens, it appears as if the CHIRs, which are KIR-related, have taken over many of the functions performed by the classical receptors in mammals.

Ig-domain containing molecules have experienced a remarkable increase in numbers during vertebrate evolution. They have become part of many vital biological processes with a particular important role in immunity and the central nervous system. One interesting possibility is that whole genome duplications, so called tetraploidizations, have been a key component in their expansion and thereby have also been central for the evolution of both FcRs and NK cell receptors [51]. The question of the role of tetraploidizations for the origin of the FcR, the LRC and related loci has been addressed in detail by Zucchetti et al, where they, by analysis of a primordial locus in tunicates, trace the expansion and diversification of the putative four copies of this locus in mammals including humans [47]. The early locus in the vase tunicate *Ciona intestinalis*, which pre-dates the two genome duplication that most likely occurred during early vertebrate evolution, has been broken into two and is now found on two chromosomes, 4 and 10 [47]. Paralogous regions to this original locus are now found on five or six chromosomes in humans, and not only four, possibly due to secondary translocations. The two loci representing the original locus in tunicates contains a number of Ig-domain containing molecules including the cell adhesion molecules, nectins and NCAM and molecules similar to CD66 (Ceacam), CD166, CD22 and nephrin [47]. Several of these have one or a few ITIMs in their cytoplasmic tails [47]. Interestingly, a number of these Ig-domain containing proteins in *Ciona* contain both V and C type domains, or V and I type domains, indicating that an early locus contained Ig domains of several types. The *Ciona* Ig domain containing members of this ancestral locus have between one to six Ig domains [47].

A finding that may favor a common origin of the FcR locus and the LRC is the presence of genes for the signaling components, FcεRIγ, DAP10, DAP12 and the TCR zeta chain in both chromosomal regions (Figures 2 and 3). These signaling components are structurally unrelated to the Ig-domain containing genes, which makes them good markers for such a process as they cannot have appeared as a result of a gene duplication from related genes in the chromosomal region. The FcεRIγ and TCR zeta genes are located on chromosome 1 and DAP10 and 12 are located on the same chromosome as the LRC locus in primates (Figures 2 and 3). A second finding that also supports genome duplications as a contributing factor is the close relationship in domain structure between members of both of these loci [50]. The majority of the genes in both loci contain C2 type domains as represented by both the classical FcRs for IgG and IgE, the FcRL molecules and the KIRs and LILRs [50].

Although all the domains of the KIRs, LILRs, the classical FcRs and FcRL molecules are of the C2 type, some genes in the FcR locus also contain V type domains. Interestingly, all the Ig domains of the PIGR are V type domains [21]. The single Ig domain of the FcαμR is closely related to the first domain (D1) of the PIGR [21] and the IgM receptor also contains a single Ig domain that belongs to the V type of Ig domains. The related genes in the tunicate *Ciona intestinalis* has V, C and I type domains indicating that both types of domains were present in such a locus during early chordate evolution.

The PIGR, which is of key importance for the transport of IgA and IgM over epithelial layers in mammals, has been found in all vertebrates from bony fish to humans. This receptor is probably also needed for the interaction of IgM with various immune cells and also for the transport of Igs across epithelial layers in fish. Interestingly, the PIGR in fish has only two or three Ig-like extracellular domains compared to five in mammals [38]–[41]. The second and third domains of the mammalian PIGR seems to be an addition later in vertebrate evolution, where these regions appear to be of importance for efficient transport of dimeric IgA [62]. One step in this process has been an internal duplication involving domains two and three, leading to an exon containing two domains, which is in contrast to all other Ig domains within various PIGR genes, which are encoded by a separate exon. This duplication did not appear or was later reverted in the chicken genome [63]. The massive expansion which has occurred among the PIGR genes in zebrafish is also striking. This fish species now has at least 25 closely related copies of this gene with members having either two or three Ig domains with the genes found on two different chromosomes (Figure 3). The functional significance of this massive increase in PIGRs is not known. Noteworthy is the absence of both the PIGR and the FcRL receptors in cartilagous fish. This indicates that both of these receptors appeared with the bony fish.

Evidence from the mammalian PIGR indicates that the J chain structure is important for efficient transport over epithelial layers of IgA and IgM [2], [21]. Interestingly, the J chain has been found in cartilagous fish and in lungfish but seems to be absent in most bony fish [2], [64]. However, despite an apparent lack of the J chain in the PIGR of carp, IgM transport at numerous epithelial sites in this fish species seems to be active [39]. IgM is present essentially in the same form in all vertebrates, and is probably the ancestor of all other isotypes. The fact that the PIGR is one of the first genes within the FcR locus to appear during vertebrate evolution may therefore seem logical.

The PIGR seems to be one of the first receptors for IgM to appear during vertebrate evolution. However, what is the origin of the other IgM receptors FcαμR and FcμR? Curiously, no evidence, even after extensive screening, has been obtained for the presence of FcαμR in fish, amphibians, birds and reptiles. The first evidence for the dual IgM/A receptor and the IgM receptor seems to be with the monotremes as one copy of each is found in the platypus genome, which indicates that these receptors appeared during early mammalian evolution. The FcαμR is the receptor that is most closely related to the PIGR, particularly to the ligand binding D1 domain of the PIGR, which is of the V type Ig domains, clearly indicating a common origin [21], [65]. The IgA/M receptor has only one Ig domain and a stalk structure that is extensively glycosylated, which makes it unique among these receptors [21], [65]. The IgM receptor also has only one Ig domain and this is of the V type.

The question then arises about the origin of these V and C2 type domain receptors. Are they products of gene duplications from an early two or three domain receptor containing both V and C2 type domains, or possibly primordial genes having only one type of domain? The domain structure of the PIGR and the FcRL molecules differs quite significantly, which may indicate a different evolutionary origin, favoring the second alternative. Conversely, the presence of receptors in tunicates, having V, C and I type domains, may instead favor the first alternative [47].

The question of the origin of the IgA receptor is also interesting as this receptor is only found in placental mammals and located in the LRC locus together with KIRs and LILRs. The FcαRI binds its ligand at the interphase between the CH2 and CH3 of human IgA, which also differs from the binding region of the classical FcRs of mammals, which binds IgE in the upper part of the CH3 domain and IgG in the upper part of the CH2 domain [22]. The chicken IgY receptor, CHIR-AB1, also binds IgY in a similar position as the IgA receptor [66]. Both FcαRI and CHIR-AB1 share an additional similar feature having a positively charged amino acid in the transmembrane region, which is involved in the interaction with the signaling component the FcεRIγ or DAP12 [49]. Therefore, the most likely explanation is that it has its origin from a regional duplication involving KIRs or LILRs, which like the IgY receptors in chickens, has gained Ig specificity.

As previously described, whole genome duplications may have had a major impact on the diversification of these receptors. However, regional duplications have most likely been equally important. Two examples are found among the IgG receptors in humans, where two new low-affinity and two new high-affinity receptors, FcγRIIC, FcγRIIIB, FcγRIB and FcγRIC, have been generated by what appears as very recent regional duplications (Figure 2). Regional duplications have also most likely been the key player in the amplification of PIGRs in zebrafish and the massive expansion of FcRL3, 4 and 5 related genes in *Xenopus* (Figure 2).

The presence of FcRL molecules, with no members of the classical FcRs in fish, indicates that FcRL molecules were among the first identifiable members of this locus. The fact that they are found in the same chromosomal region as the IgE and IgG receptors in mammals, as well as that the classical FcRs are first found in marsupials, strengthens the possibility that the FcRL molecules are the ancestors of the mammalian IgG and IgE receptors. The identification of IgA and IgG binding of FcRL4 and 5, respectively, also supports this idea [34], [35]. In addition, the phylogenetic analysis clearly favors this scenario as the mammalian IgG and IgE receptors are found in a separate branch, close to the FcRL cluster (Figure 4). Finally, there are similarities in domain structure between the IgG and IgE receptors and FcRL3, 4, 5 and A and B, indicating that the three first domains of FcγRI and the two domains of FcγRII, III and IV came from a FcRL3, 4, 5, A or B gene (Figure 5). Interestingly, an IgM receptor has been identified in the channel catfish (*Ictalurus punctatus*), which is most likely a soluble receptor as it lacks both a transmembrane region and a classical GPI anchor [67]. This molecule has three Ig domains and, in the phylogenetic analysis, is most closely related to the FcRL molecules. This latter finding further supports the notion that the classical FcRs in mammals originate from FcRL molecules [67]. Related molecules to this IgM receptor have also been found in other fish species, where non-membrane anchored soluble molecule forms also exist [67]. An important subsequent question arises about its function, as most classical receptors are cell bound, whereas these fish IgM receptors seem to be entirely soluble molecules. Their functions are a mystery as these soluble receptors do not seem to be able to signal to cells.

The initial analysis of FcRs in a non-placental mammal, the opossum, showed the presence of most of the classical receptors except the IgA, the A/M, the IgM and the high-affinity IgG receptor [24]. The presence of the two-domain receptors and the lack of the high-affinity receptor for IgG indicated that the two-domain receptors were the first IgG and IgE specific receptors to appear during mammalian evolution. However, the identification of three-domain receptors in both a monotreme, the platypus, and in an amphibian, the Western clawed frog, contested this idea. Interestingly, in the Maximum-likelihood and the Distance trees the platypus FcγRI and FcγRIL genes clustered with the classical receptors and in particular the FcγRI, indicating that these receptors in the platypus may be early representatives of the classical receptors. The isotype specificity of these two- and three-domain receptors is not known, nor whether they even bind Igs at all. They may actually be the missing link to the evolution of the complex set of receptors seen in placental mammals and the appearance of these receptors from early FcRL family members. This is an obviously very interesting area of investigation. Also of interest is that one of these three domain receptors has 2 ITIMs whereas the other has no motifs that can be classified as ITIMs or ITAMs. This receptor may therefore connect with the γ chain for signaling similar to many of the classical FcRs in placental mammals.

Interestingly, the FcRL molecules in Xenopus did, in the various trees form a separate branch in the phylogenetic tree indicating that the individual members have appeared after the separation from the other vertebrate lineages. An alternative explanation for this clustering is that there has been extensive homogenization by gene conversion within these lineages.

In summary, the analysis of the origin of the FcRs, using a large panel of vertebrate genomes, provides strong support for the notion that the complexity in FcRs has increased in parallel with the complexity of the Ig isotypes. This began with the appearance of PIGRs, the FcRL molecules and the signaling subunit, the γ chain. These three receptors/receptor families seem to have appeared with the bony fish. The end result of this expansion is a complex set of receptors for almost all the different isotypes including three to four for IgG, one for IgE, one for IgA, one for IgM and a shared receptor for IgM and IgA. The appearance of the various Ig receptors also seems to have taken different routes in different vertebrate lineages. In mammals, most of the FcRs have developed from the FcRL molecules of the FcR locus, whereas in birds, multiple IgY receptors have developed within the LRC locus. Although distantly related genes to the LRC locus in mammals are found in tunicates, clearly identifiable homologs to the LCR locus genes of mammals with only C2 domains are also first seen in bony fish, indicating a major evolutionary event concerning the evolution of FcRs and LRC genes at the base of the bony fish. The first solid evidence for a direct homolog to the classical FcRs is found among the two-domain receptor homologs in marsupials. However, the three domain receptors in the platypus also clustered with the classical receptors in the Maximum-likelihood and Distance trees indicating that they may be early ancestors of the subgroup of FcRL genes that formed the classical receptors. The close structural relationship between FcRL molecules and in particular FcRL3, 4, 5, A and B involving domain types D1, D2 and D3 also provides strong support for FcRL molecules being the direct ancestors of the various classical IgG and IgE receptors in marsupials and placental mammals. A second major step in the evolution of FcRs may therefore have occurred in early mammals when one or a few FcRL genes formed the ancestor of the classical FcRs and also sequences from the PIGR duplicated and became an early ancestor of the IgA/M and IgM receptors. The analysis of the possible isotype specificity of the various related receptors in platypus may shed important light on the origin of the classical IgG and IgE receptors in placental mammals. This may also give clues to the origin of the role of IgE in triggering mast cell granule release through its interaction with IgE specific Fc receptors (work in progress).

## Supporting Information

Supporting Information S1 **Accession numbers for all genes used in the alignment figures.**
(DOCX)

Figure S1 **Clustal X NJ tree.** The rooted neighbor-joining tree of vertebrate Fc receptors was analyzed in Clustal X with 100 replicates.
(EPS)

Figure S2 **Phylip ML tree.** The rooted Maximum-likelihood tree of vertebrate Fc receptors was calculated using likelihood parameters with 100 replicates using in Phylip programs. Node bootstrap values are indicated.
(EPS)

Figure S3 **Phylip distance tree.** The rooted distance matrix tree of vertebrate Fc receptors was calculated using likelihood parameters with 100 replicates using in Phylip programs. Node bootstrap values are indicated in the phylogenetic tree.
(EPS)

Figure S4 **MEGA Domain Tree.** The individual domains of all receptors shown in figure 5 were analyzed individually for their sequence relatedness. The individual domain types were color coded with the same colors as in figures 5. The rooted maximum likelihood tree was constructed by using MEGA5.2 soft wear. Bootstrap analysis was performed with 500 replicates. Bootstrap values are indicated in the phylogenetic tree.
(EPS)

Figure S5 **Domain structure of a number of additional FcRL molecules from the frog (Xenopus) not presented in** **figure 5** **.** A marked difference in the presence of ITIMs is observed among the various FcRL members in frog. Some have up to 2 consensus ITAMs whereas many have not got a single canonical site or distantly related site.
(EPS)
