# Correction: Fc Receptors for Immunoglobulins and Their Appearance during Vertebrate Evolution

**DOI:** 10.1371/journal.pone.0124530

**Published:** 2015-04-01

**Authors:** The PLOS ONE Staff

Fig 5 is incorrect. The authors have provided a corrected version here.

**Fig 5:**
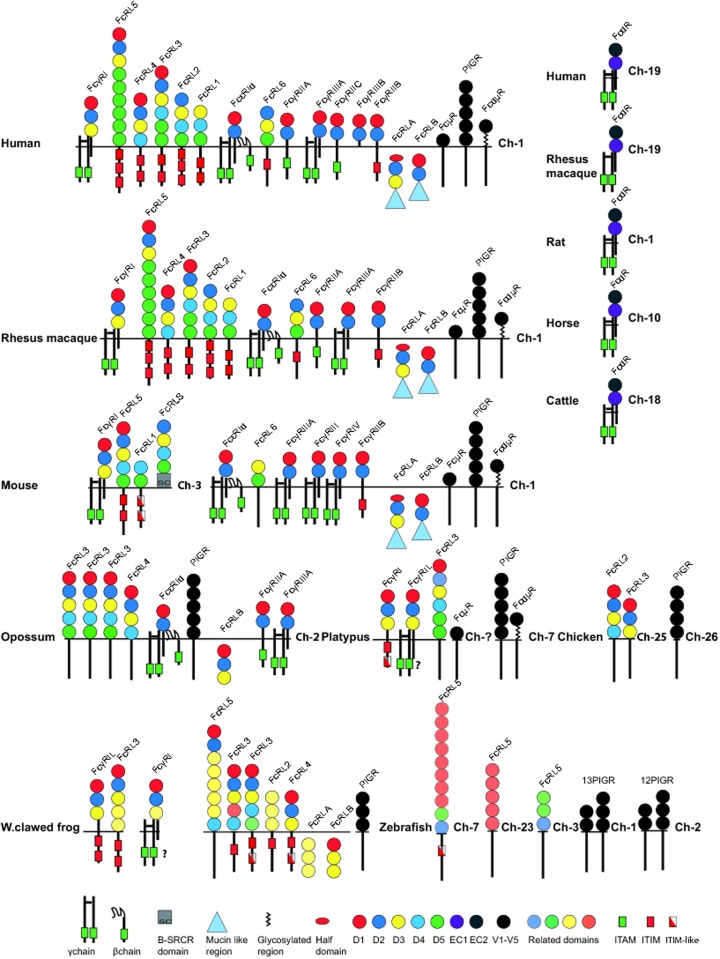
A summary of domain structures and signaling motifs of the various vertebrate Fc receptors. The Ig-like domains are depicted as filled circles with color-coding according to the similarities in sequence based on phylogenetic analyses [24], [58]. The domain type D1, D2, D3, D4 and D5 show a relatively conserved pattern in most tetrapods and have therefore been color-coded in red dark blue, yellow, light blue and green. A phylogenetic analysis of all the individual domains presented in Fig 5 and a few additional receptors are presented in supplementary figure S4. The color-coding in Fig 5 is based on this supplementary figure. The extracellular regions, the transmembrane regions and cytoplasmic tails are not to scale in order to show the positions of potential signaling motifs like ITAMs (green boxes) and ITIMs (red boxes), which regulate the biological function the Fc receptors. Non-consensus ITIMs are also indicated as boxes with half red half white. Some of the intracellular proteins contain C-terminal mucin-like regions that are depicted as blue triangles.

S5 Fig is incorrect. The authors have provided a corrected version here.

## Supporting Information

S5 Fig Domain structure of a number of additional FcRL molecules from the frog (Xenopus) not presented in fig 5. A marked difference in the presence of ITIMs is observed among the various FcRL members in frog. Some have up to 2 consensus ITAMs whereas many have not got a single canonical site or distantly related site.
(TIF)
