# Supplementary material for: Fc Receptors for Immunoglobulins and Their Appearance during Vertebrate Evolution
Source: PLoS One. 2014 May 9;9(5):e96903. doi: 10.1371/journal.pone.0096903 (PMC4016189; doi:10.1371/journal.pone.0096903)
Supplement: Supporting Information S1 — Accession numbers for all genes used in the alignment figures. (DOCX) [file pone.0096903.s001.docx]

**SUPPORTING INFORMATION S1**

The gene accession numbers used in constructing figures 2-5 and S1-S4 are listed here below.

**Human** (*Homo sapiens*); FcγRIB (NM_001244910), FcγRIC (NR_027484), FcγRIA  FcRL5 (NM_001196388), FcRL4 (NM_031282), FcRL3 (NM_052939), FcRL2 (NM_030764), FcRL1 (NM_052938), FcεRIα  FcRL6 (NM_001004310), FcεRIγ (NM_004106), FcγRIIA (NM_001136219), FcγRIII NM_001127596 FcγRIIC (NM_201563), FcγRIIIB (NM_00057 FcγRIIB (NM_001002274), FcRLA (NM_001184866), FcRLB (NM_001002901), FcμR (FAIM3) (NM_005449), PIGR (NM_002644), FcαμR (NM_032029) and FcαR (NM_002000), TCRZ (NM_000734). **Rhesus macaque** (*Macaca mulatta*); FcγRIII (NM_001048248), FcγRIIA (NM_001257300), FcγRIIB (NM_001271648), FcγRI (NM_001257304), FcεRIα (XM_00117218), FcεRIγ (NM_001265840), FcαR (NM_001039950), FcμR (FAIM3) (NM_001258068), FcαμR (XR_009842), PIGR (XM_001083307), FcRL1 (XM_001116929), FcRL2 (XM_001116902), FcRL3 (XM_002801864), FcRL4 (XM_001116859), FcRL5 (XR_014533), FcRL6 (XM_001117280), FcRLA (XM_001118111) and FcRLB (XM_001118141). **Chimpanzee** (*Pan troglodytes*); FcγRIIIA (XM_001174057), FcγRIIIB (XM_001159557), FcγRIIA (NM_001009077), FcγRIIB (XM_001165641), FcγRI (XM_001165641), FcεRIα XM_002809952), FcμR (FAIM3) (XM_003308711), FcαμR (XM_001165644), PIGR (XM_003308710), FcRL1 (XM_001168778), FcRL2 (XM_524903), FcRL3 (XM_001168488), FcRL4 (XM_001168141), FcRL5 (XM_524901), FcRL6 (XM_524928), FcRLA (XM_003308571), FcRLB (XM_001174174). **Orangutan** (*Pongo abelli*); FcγRIIIA XM_002809883 FcγRIIA (XM_002809886), FcγRIIB (XM_002809881), FcγRI (XM_002834824), FcεRIα XM_001170670),  FcεRIγ (XM_002809891), FcαR (XR_095724), FcμR (FAIM3) (XM_002809552), FcαμR (NM_001131375), PIGR (NM_001131626), FcRL1 (XM_002809991), FcRL3 (XM_002809993), FcRL5 (XM_002809992), FcRL6 (XM_524928), FcRLA (XM_003308571), FcRLB (XM_001174174). **Dog** (*Canis lupus familaris*); FcγRIIIA (XM_536141), FcγRIIB (XM_003640212), FcγRI (NM_001002976), FcεRIα (NM_001110766), FcεRIγ (NM_001003171), FcμR (FAIM3) (XM_005622301), FcαμR (XM_005622953), PIGR (XM_537133), FcRL1 (XM_005622662), FcRL2 (XM_005622661), FcRL3 (XM_005622976), FcRL4 (XM_005622977), FcRL5 (XM_005622663), FcRL6 (XM_003640204), FcRLA (XM_005640867), FcRLB (XM_0056640941). **Cattle** (*Bos taurus*); FcγRIIIA (NM_001077402), FcγRIIB (NM_174539), FcγRIIC (NM_001109806), FcγRI (NM_174538), FcεRIα (NM_001100310), FcεRIγ (NM_174537), FcαR (NM_001012685), FcμR (FAIM3) (XM_002693895), FcαμR (XM_002701487), PIGR (NM_174143), FcRL1 (NM_001102045), FcRL2 (XM_003581924), FcRL3 (XM_002685955), FcRL4 (XM_614632), FcRL5 (XM_003585824), FcRL6 (XM_002685897), FcRLA (XM_001251523), FcRLB (NM_001192565). **Pig** (S*us scrofa*); FcγRIIIB (NM_214391), FcγRIIB (NM_001033013), FcγRI (NM_001033011), FcεRIα (XM_001929139), FcεRIγ (NM_001001265), FcαR (NM_001123112), FcαμR (XM_003357420), PIGR (NM_214159), FcRL1 (XM_001927548), FcRL3 (XM_003125692), FcRL4 (XM_001927558), FcRL5 (XM_003125693), FcRL6 (XM_003481428), FcRLA (XM_003484244), FcRLB (XM_001925958). **Rabbit** (*Oryctologus cunicullus*); FcγRIIIA (XM_002715247), FcγRIIB (XM_002715246), FcγRI (XM_002715373), FcεRIα (XM_002715280), PIGR (NM_001171045), FcRL1 (XM_002715302), FcRL2 (XM_002715303), FcRL4 (XM_002715305), FcRL5 (XM_002715306), FcRL6 (XM_002715274), FcRLA (XM_002715125), FcRLB (XM_002715245). **Mouse** (*Mus musculus*); FcγRIV (NM_144559), FcγRIII (NM_010188), FcγRIIB (NM_010187), FcγRI (NM_010186), FcεRIα (NM_010184), FcεRIγ (NM_010185), FcμR (FAIM3) (NM_026976), FcαμR (NM_001170632), PIGR (NM_011082), FcRL1 (NM_153090), FcRL5 (NM_183222), FcRLS (NM_030707), FcRL6 (NM_001164725), FcRLA (NM_001160215), FcRLB (NM_001029984). **Rat** (*Rattus norvegius*); FcγRIIIA (NM_207603), FcγRIIA (NM_053843), FcγRIIB (NM_001135992), FcγRI (NM_001100836), FcεRIα (NM_012724), FcεRIγ (NM_001131001), FcαR (NM_201992), FcμR (FAIM3) (NM_001014843), FcαμR (XM_001060181), PIGR (NM_012723), FcRL1 (XM_001058249), FcRL3 (XM_001058446), FcRLS (NM_001107702), FcRLA (NM_001100682), FcRLB (NM_001191931). **Horse** (*Equus caballus*); FcεRIα (NM_001099767), FcεRIγ (XM_001503798), FcαR (NM_001099764), PIGR (XM_001492298), FcRL3 (XM_003365049), FcRL3L (XM_003365048), FcRL3L (XM_001489001), FcRL5L (XM_001915537), FcRL6 (XM_001491018), FcRLA (XM_001488095), FcRLB (XM_005609919). **Opossum** (*Monodelphis domestica*); FcεRIγ (XM_001381663), PIGR (XM_001366104), FcRL3L (XM_001380068), FcRL4L (XM_001380038), FcRL3L (XM_001380061), FcRL3L (XM_001380083), FcRLB (XM_001369362). **Platypus** (*Ornithorhynchus anatinus*); FcγRI (XM_001521652), FcγRIL (XM_001521660), FcRL3 (XM_001515313), FcμR (XM_001518104), FcαμR (XM_001508674), PIGR (XM_001508602). **Chicken** (*Gallus gallus*); FcRL2 (NM_001097529), FcRL3 (XM_004948316), PIGR (NM_001044644). **Turkey** (*Meleagris gallopovo*); FcRL4 (XM_003212776). **Zebra finch** (*Taeniopygia guttata*); PIGR (XM_004177123). **Green anole** (*Anolis carolinennsis*); PIGR (XM_003224013). **Western clawed frog** (*Xenopus* (*silunarana*) *tropicalis*); FcγRI; (XM_002938341), (NM_001114208), (BV088875), FcγRII (XM_004917529), FcRL2; (XM_002940689), (XM_002943141), (XM_002942887), FcRL3; (XM_004919193), (XM_002940692), (XM_002940690), (XM_002940476), (002942254), (XM_002943398), (XM_002941145), (XM_004917419), (XM_002942254), FcRL4; (XM_002940693), (XM_002943399), (XM_002942794), (XM_004917595), (XM_002938998), (XM_004919342), FcRL5; (XM_004917418), (XM_002940479), (XM_002943602), (XM_002943603) (XM_002943414), (XM_002942258), (XM_004919192), (XM_002942886), (XM_002942257), (XM_002940480), (XM_002934330), (XM_004919977), FcRLA (XM_004917529), FcRLB (XM_002941146), PIGR; (XM_002932134), (XM_004911391). **Zebra fish** (*Danio rerio*); FcεRIγ (NM_001100156), PIGR; (XM_005160234), XM_005160224), (XM_005163603), (XM_001921914), (XM_001921704), (XM_005163604), (XM_005160238), (XM_001343321), (XM_002664524), (XM_005169739), (XM_001921906), (XM_005169733), (XM_005169737), (XM_001340936), (XM_002660938), (XM_005169734), (XM_005163605), (XM_003197957), (XM_002660949), XM_003197954), XM_00516023), (XM_005160231), XM_001921723), XM_005160236), NM_001083878), XM_005160233).
