# Supplementary figures and images for: Correction: Fc Receptors for Immunoglobulins and Their Appearance during Vertebrate Evolution
Source: PLoS One. 2015 Apr 1;10(4):e0124530. doi: 10.1371/journal.pone.0124530 (PMC4382169; doi:10.1371/journal.pone.0124530)

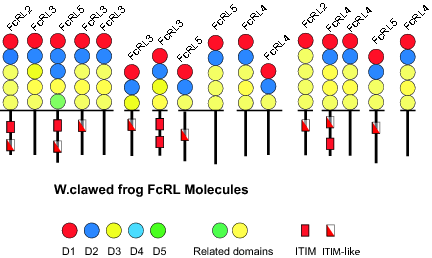

Supplement: S5 Fig — A marked difference in the presence of ITIMs is observed among the various FcRL members in frog. Some have up to 2 consensus ITAMs whereas many have not got a single canonical site or distantly related site. (TIF) [file pone.0124530.s001.tif]
